# Distribution of Airborne Fungi in Vehicles and Its Association with Usage Patterns

**DOI:** 10.3390/jof11100725

**Published:** 2025-10-10

**Authors:** Raúl Asael Rodríguez-Villarreal, Mariana Elizondo-Zertuche, Nydia Orué-Arreola, Juan Adame-Rodríguez, Larissa E. Gordillo-Mata, Miguel González-Enríquez, Brandon Ortega-Castillo, Patricio Adrián Zapata-Morín, Efrén Robledo-Leal

**Affiliations:** 1 Laboratorio de Micología y Fitopatología, Departamento de Microbiología e Inmunología, Facultad de Ciencias Biológicas, Universidad Autónoma de Nuevo León, San Nicolás de los Garza, Nuevo León 66455, Mexico; raul.rodriguezvill@uanl.edu.mx (R.A.R.-V.);; 2 Departamento de Microbiología, Facultad de Medicina, Universidad Autónoma de Nuevo León-Francisco I. Madero & Dr. Eduardo A. Pequeño, Mitras Centro, Monterrey 64460, Mexico

**Keywords:** conidia, mycosis, pollution, Monterrey, bioaerosols

## Abstract

Airborne fungal exposure in confined indoor environments is a growing public health concern, however the microbial composition of air inside private vehicles remains underexplored. This study aimed to characterize culturable airborne fungi in vehicle cabins and evaluate their association with environmental and behavioral variables. Air samples (100 L) were collected from 69 vehicles using a standardized culture-based method. Simultaneously, a detailed survey was administered to vehicle owners to document usage patterns, maintenance habits, and odor perception. Results revealed a total culturable fungal load of 31,901 CFU/m^3^, with *Cladosporium*, *Aspergillus*, and *Penicillium* as the most frequently isolated genera. Statistical analysis showed that fungal abundance and community composition were significantly associated with vehicle usage factors such as air disturbance, parking environment, air filter maintenance, and perception of musty odors. Vehicles parked outdoors had significantly higher *Bipolaris* levels, while lack of regular filter replacement was strongly associated with elevated *Alternaria* abundance. The presence of musty or moldy odors correlated with a 2.5-fold increase in *Aspergillus* levels. Redundancy analysis confirmed that odor perception and parking behavior were the strongest predictors of fungal community structure, with specific genera displaying distinct ecological preferences across usage conditions. Usage patterns and maintenance habits significantly influence in-cabin fungal communities, with implications for respiratory health, particularly due to the presence of allergenic and opportunistic genera like *Aspergillus*, *Alternaria*, and *Bipolaris*. Regular air filter maintenance and attention to odor cues may help reduce fungal load and associated health risks.

## 1. Introduction

The metropolitan area of Monterrey (Nuevo León, México) ranks as the fourth most traffic-congested city in the country, with an average of 51 h lost per person in traffic every month [1]. As a result, residents spend a substantial portion of their daily lives inside vehicles, often with windows closed due to extreme outdoor temperatures. This prolonged confinement leads to continuous exposure to the vehicle’s indoor air environment, a space whose microbial quality remains largely unexamined.

Indoor air is known to harbor a complex mix of biological particles, including fungal spores that may adversely affect respiratory health [2]. Although previous studies have shown that vehicular traffic contributes to elevated fungal particle levels in outdoor air [3] little is known about how these particles infiltrate or persist in the enclosed cabin air of private vehicles.

The urgency of addressing fungal exposure has gained prominence since the World Health Organization published its first-ever Fungal Priority Pathogens List [4], classifying medically important fungi such as *Aspergillus*, *Fusarium*, *Scedosporium*, Mucorales, and several yeasts into threat tiers based on multidimensional risk criteria. Several of these genera have been previously detected by our group in both indoor and outdoor air samples from Monterrey [5].

Vehicle interiors represent a unique and understudied microenvironment. HVAC systems and air filters can act as reservoirs for airborne fungi, especially filamentous species like *Aspergillus*, which are capable of surviving and proliferating in filter media [6,7]. Infrequent maintenance or aging filters can further exacerbate fungal loads within the cabin, while variables such as ventilation mode and seasonality modulate exposure levels [4,8].

Despite accumulating evidence on fungal contamination in the air inside vehicles [6,7,8], few studies have examined how behavioral and usage patterns—such as frequency of use, passenger occupancy, air filter maintenance, or ventilation habits—affect in-cabin fungal composition. This knowledge gap is particularly acute in Mexico.

Although there is an increasing amount of data about airborne fungi inside vehicles (6, 7, 8], a holistic look at how vehicle usage variables e.g., frequency of trips, ventilation habits, occupancy, and maintenance routines, influence airborne fungal diversity remains limited, especially in Mexico. Therefore, to better assess the potential health risks associated with daily exposure to air inside vehicles, the present study aims to answer the question: What is the in-cabin airborne fungal concentration and diversity, and how is it correlated with usage variables? This integrated approach addresses a critical knowledge gap by linking real-world vehicle usage patterns with fungal community dynamics, offering insights that are directly relevant to public health and cabin maintenance practices.

## 2. Materials and Methods

### 2.1. Area of Study

**Figure 1:**
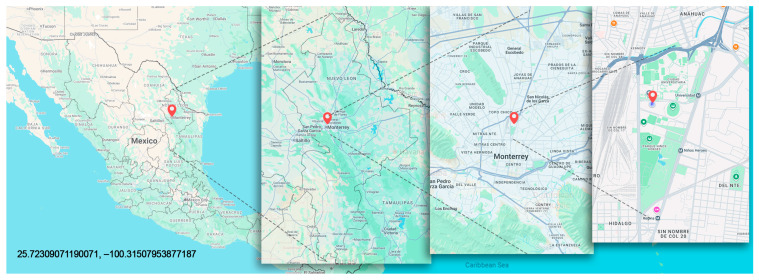
Geographical map of the sampling site (Google Maps); red mark indicates sampling location.

The sampling was conducted in the main parking area of the Universidad Autónoma de Nuevo León (UANL; Figure 1), the third largest university in Mexico and the largest public institution in the northern region of the country.

### 2.2. Survey Design

A 21-item survey was administered to collect information on vehicle usage habits, environmental exposure, interior materials and fabrics, cleaning and maintenance routines, and user perception of mold presence. Additionally, it was recorded whether the vehicle was arriving or departing at the time of sampling, as a proxy for distinguishing between disturbed and undisturbed in-cabin air conditions, respectively. The survey questions were phrased to elicit either binary responses or ratings on a Likert scale, facilitating the numerical conversion of responses for subsequent statistical analysis (see Supplementary Material).

### 2.3. Sampling Method

Vehicle owners were approached on site and invited to voluntarily participate in a survey regarding their vehicle usage patterns. Following the completion of the survey, a 100-L air sample was collected from the interior of each vehicle, under standardized conditions: all doors and windows were closed, and the engine was turned off.

Air samples were collected using an AirTest^®^ device (LCB Food Safety, Boz, France). Following previously established methods [5,9], 100 L of air were impacted onto Petri dishes containing Rose Bengal–malt extract agar (RBME; BD, USA). This medium was selected for its effectiveness in cultivating airborne fungi: malt extract supports airborne fungal growth [10], while rose bengal inhibits rapid radial expansion, reducing colony overlap and improving quantification and morphological observation. The sampling device was placed on the front passenger seat, and the plates were immediately incubated at 25 °C.

Air samples were taken using an AirTest^®^ device (LCB food safety, France). Following the methods of previous reports [5,9] 100 L of air was impacted onto Petri dishes containing Rose Bengal-malt extract-agar (RBME; BD, USA). This medium is used due to malt extract’s suitability for airborne fungi cultivation and rose bengal slows down fungal radial growth, avoiding rapid colony overlaying thus allowing for better quantitation and observation. The sampling device was located at the front passenger seat and plates were incubated at 25 °C immediately after.

### 2.4. Fungal Quantitation and Identification

Beginning on day 5 of incubation, colony forming units (CFU) were quantified according to manufacturer’s instructions, counting the CFUs and extrapolating them to the concentration per cubic meter of air, according to the device’s CFU reading table (which combines most probable number and Fellers statistical correction). Fungal genera were identified based on their macroscopic and microscopic morphological features [11]. Fungi that did not sporulate after 30 days were reported as sterile mycelium. Yeasts were considered a group on their own because morphological identification is not feasible.

### 2.5. Statistical Analysis

All analyses were conducted using R version 4.3.0 [12]. The following R packages were used: dplyr (v1.1.3), ggplot2 (v3.4.2), ggpubr (v0.6.0), rstatix (v0.7.2), car (v3.1-2), broom (v1.0.5), readr (v2.1.4), stringr (v1.5.0), tidyr (v1.3.0), RColorBrewer (v1.1-3), and ggcorrplot (v0.1.4).

The abundance of each fungal species was analyzed individually and in combination, and the effects of questionnaire variables were evaluated through univariate and multivariate statistical methods.

#### 2.5.1. Data Preprocessing

Fungal abundance data were filtered to retain only rows with non-missing, non-zero CFU values. For each fungal species, a new variable was generated by applying a base-10 logarithmic transformation:

log10(CFU)=log10(Yi),Yi>0

#### 2.5.2. Univariate Analysis

To assess non-parametric relationships between individual questionnaire variables and fungal abundance, two primary tests were applied:

When a variable had exactly two levels, the Mann–Whitney U test (Wilcoxon rank-sum) was used to compare CFU distributions across groups:

$$U=min(U_{1}\;,U_{2}\;),U_{i}\;=R_{i}\;-\frac{n_{i}\;(n_{i}\;+1)}{2}\;$$

Effect size was reported using the rank-biserial correlation:

$$r=\frac{Z}{\sqrt{N}}$$

where Z is the standardized test statistic and N is the total sample size.

For questionnaire variables with more than two levels, the Kruskal–Wallis test was applied to detect differences in distributions:

$$H=\frac{12}{N\left(N+1\right)}=\sum_{i=1}^{g}{n_{i}\left(R_{i}-\bar{R}\right)}^{2}$$

Effect size was expressed as eta-squared:

$$\eta 2=\frac{H}{N-1}$$

Post hoc pairwise comparisons between groups were performed using pairwise Wilcoxon rank-sum tests.

#### 2.5.3. Correlation Analysis

To examine monotonic trends between questionnaire variables and fungal abundance, Spearman’s rank correlation coefficient (ρ) was computed:

$$\rho =1-\frac{6\sum d_{i}^{2}}{n(n^{2}-1)}$$

where $d_{i}$ is the difference between ranks for observation i, and n is the number of observations.

#### 2.5.4. Multivariate Modeling (GLM)

To evaluate the combined effects of questionnaire variables on fungal abundance, generalized linear models (GLMs) were constructed for each species. The models used a Gaussian family and identity link function, with the log-transformed CFU as the response variable:

$${log}_{10}{\left(CFU\right)}_{i}=\beta_{0}+\sum_{j=i}^{k}\beta_{j}X_{ij}+\varepsilon_{i}$$

where $\beta_{0}$ is the intercept, $\beta_{j}$ are coefficients for predictor variables $X_{ij}$ and $\varepsilon_{i}$ are residual errors.

Each categorical predictor was coded using dummy variables, with the lowest level used as the reference. Coefficient estimates were transformed to fold-change values for interpretation:

$$Fold\;Change=10^{\beta_{j}}$$

Models with fewer than 10 valid observations were excluded to ensure robustness. Coefficients, standard errors, test statistics, and *p*-values were extracted using the broom::tidy() function. Variables with *p* < 0.05 were considered statistically significant.

#### 2.5.5. Redundancy Analysis (RDA)

To assess how the full fungal community structure was constrained by questionnaire-derived variables, redundancy analysis (RDA) was conducted on a log-transformed species matrix:

$${log}_{10}(CFU)={log}_{10}(Y_{i}+1),Y_{i}\geq 0$$

where $Y_{i}$ is the CFU count of species i The explanatory matrix included questionnaire variables treated as categorical factors. The RDA model was specified as:

Species matrix∼Questionnaire variables

Variance explained by each RDA axis was computed, and an ANOVA with 999 permutations was used to assess the significance of each explanatory variable.

#### 2.5.6. Relative Abundance Analysis

To further examine species-level responses to questionnaire variables, relative abundance was computed for each species within each vehicle:

$${Relative\;Abundance}_{ij}=\frac{{CFU}_{ij}}{\sum_{k=1}^{S}{CFU}_{ik}}$$

where ${CFU}_{ij}$ is the abundance of species j in sample i and S is the total number of species.

Only species-variable pairs with *p*-values < 0.05 were reported.

## 3. Results

**Figure 2:**
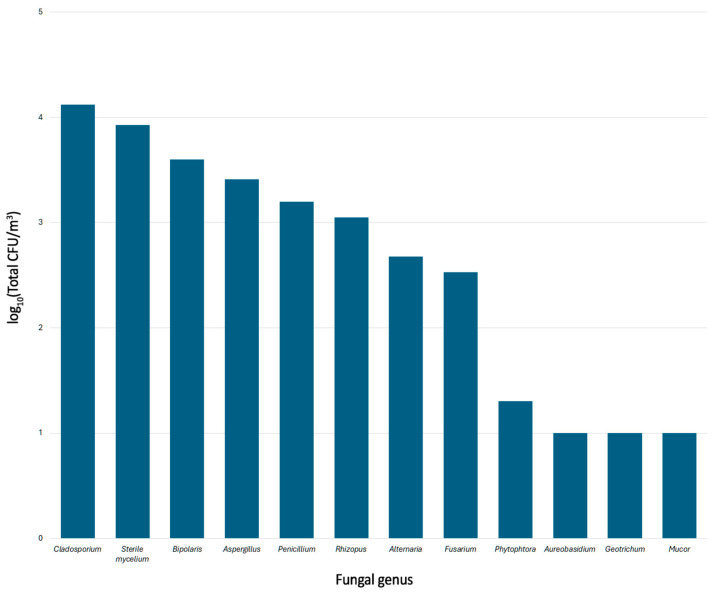
Distribution of isolated fungal genera (global).

A total of 69 vehicles were sampled. The global amount of culturable fungal CFU/m^3^ recovered was 31,901; the genera distribution is shown in Figure 2. The most frequently isolated genera included *Cladosporium*, *Aspergillus* and *Penicillium*, with *Cladosporium* showing the highest CFU count, followed by sterile mycelium group and *Bipolaris*.

### 3.1. Univariate Analysis

Across the 21 item-derived variables, statistical comparisons using Mann–Whitney U tests and Spearman correlation revealed significant associations between disturbed and undisturbed air, parking outdoor vs. indoor, regular air filter change and the perception of musty/moldy odors inside the vehicle.

**Figure 3:**
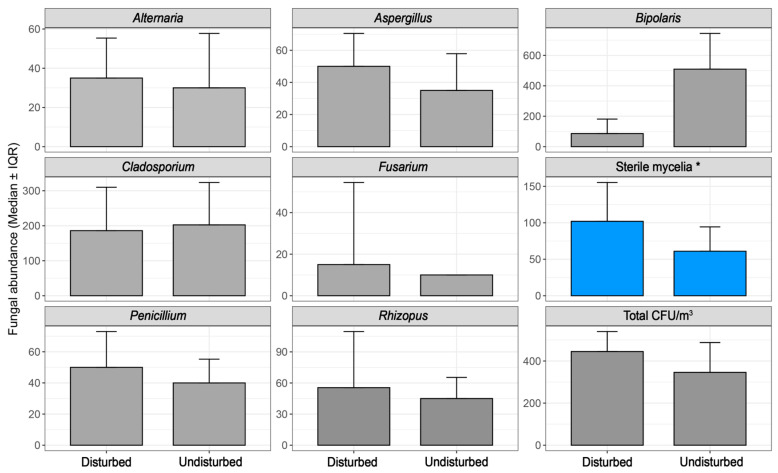
Effect of air disturbance on in-cabin fungal abundance. * refers to variables with statistical difference (*p* < 0.05).

Regarding disturbed and undisturbed cabin air, a weak but significant Spearman correlation (ρ = −0.27, *p* = 0.035) and a significant Mann–Whitney U result (*p* = 0.037, r = 0.27, direction: 1 > 2) was found between disturbed air and sterile mycelium. This was further supported by generalized linear modeling (GLM), which found that this behavioral factor was associated with a ~49% reduction in CFU levels (fold change = 0.51, *p* = 0.011) when vehicles were about to leave when sampled (i.e., undisturbed air). This is relevant since sterile mycelium, as a group, represented the second most abundant fungal group recovered in this study (Figure 3).

**Figure 4:**
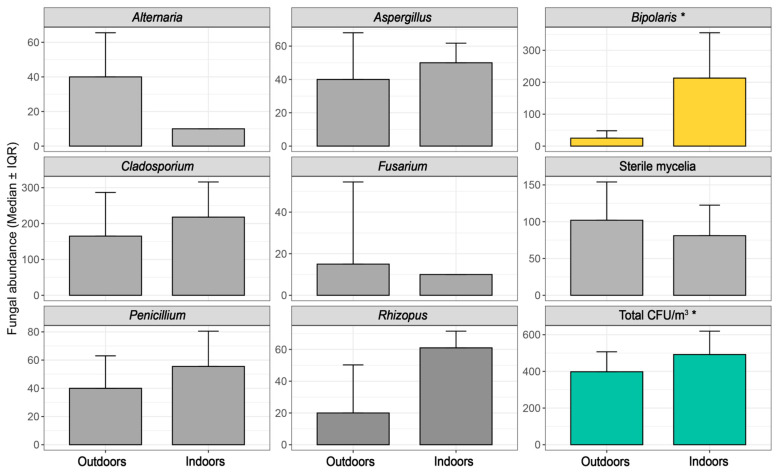
Effect of parking environment on in-cabin fungal abundance. * refers to variables with statistical difference (*p* < 0.05).

**Figure 5:**
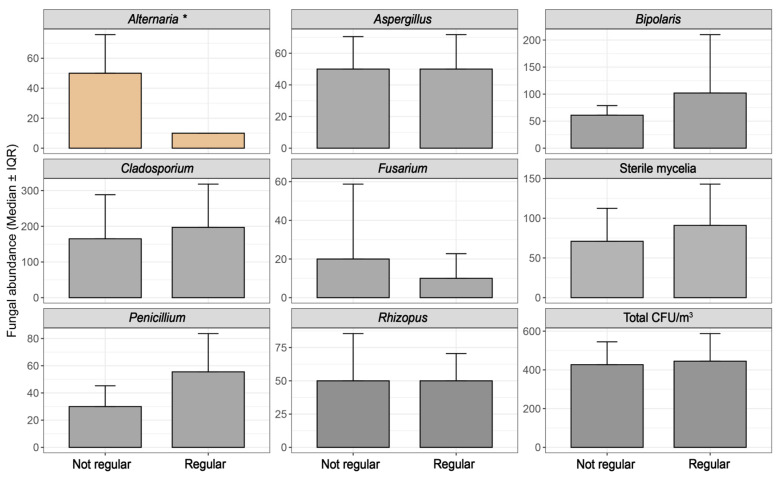
Impact of air maintenance on in-cabin fungal abundance. * refers to variables with statistical difference (*p* < 0.05).

Parking location (survey item 2.1) was significantly associated with the abundance of *Bipolaris* (ρ = 0.67, *p* = 0.0023; MWU *p* = 0.0066, r = −0.65, direction: 1 > 0) and total CFU load (ρ = 0.25, *p* = 0.042; MWU *p* = 0.043, r = −0.24). GLM results revealed a substantial ~19-fold increase in *Bipolaris* CFU in vehicles parked in open spaces compared to those parked indoors overnight (Table S1), highlighting the role of parking behavior in fungal accumulation dynamics and a putative impact in allergies (Figure 4). Similarly, air filter replacement (survey item 4.3) was significantly associated with *Alternaria* abundance. Spearman correlation indicated a strong negative relationship (ρ = −0.71, *p* = 0.023), and the Mann–Whitney U test confirmed that vehicles without regular air filter maintenance exhibited significantly higher *Alternaria* levels (*p* = 0.046, effect size r = 0.65, direction: 0 > 1; Figure 5).

**Figure 6:**
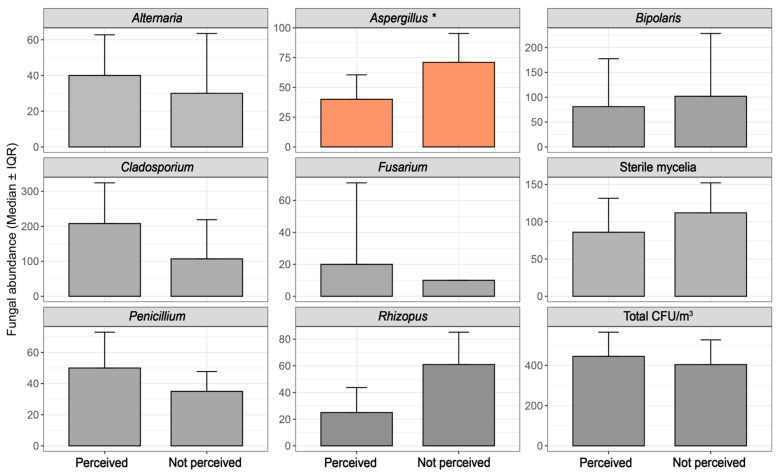
Impact of musty/moldy odor perception on in-cabin fungal abundance. * refers to variables with statistical difference (*p* < 0.05).

A notable correlation was observed between perceived musty/moldy odor inside vehicles and fungal presence (survey item 5.1). A positive answer was significantly associated with higher *Aspergillus* abundance, as confirmed by Spearman correlation (ρ = 0.44, *p* = 0.006) and Mann–Whitney U test (*p* = 0.008, effect size r = −0.43, direction: 1 > 0). The corresponding GLM result also confirmed this trend, indicating that the presence of odor was associated with a ~2.55-fold increase in CFU abundance (*p* = 0.0072). Median CFU levels were clearly elevated in odor-reported vehicles (Figure 6), reinforcing moisture perception as a potential indicator for *Aspergillus* proliferation.

### 3.2. Redundancy Analysis (RDA)

**Figure 7:**
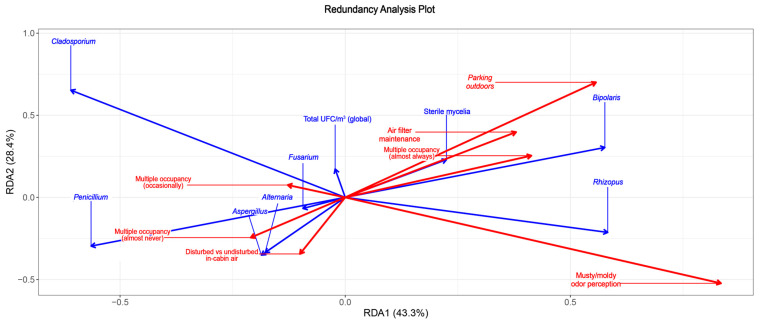
Redundancy analysis plot. Greater distance and opposite direction of items reflect greater independence. Blue refers to fungal genera; red refers to vehicle use variables.

Redundancy analysis showed that the constrained survey variables explained a substantial proportion of the variance in fungal community composition, with the first two RDA axes accounting for 43.3% (RDA1) and 28.4% (RDA2) of the variation (Figure 7).

Among the explanatory variables, musty/moldy smell perception remained the strongest and only highly significant predictor of community composition (ANOVA, *p* = 0.001), confirming its central role in driving fungal differentiation across vehicles. Additionally, parking environment emerged as a significant contributor (*p* = 0.033), underscoring this variable’s influence on the composition and abundance of key fungal genera. Other variables did not independently explain significant variation but contributed contextually to the multivariate structure.

The species scores and biplot vectors revealed clear ecological gradients *Aspergillus*, *Alternaria*, and *Penicillium* aligned negatively along RDA1 and RDA2, suggesting potential sensitivity to conditions associated with odor detection and enclosed or filtered environments. In contrast, *Bipolaris* and *Rhizopus* clustered positively along RDA1 and RDA2, showing strong associations with parking environment and musty/moldy smell perception, consistent with prior GLM findings suggesting their proliferation under more exposed or moisture-prone conditions. Sterile mycelia displayed moderate alignment with these two variables, suggesting it may also be favored under similar microclimatic regimes. Meanwhile, *Cladosporium* was notably separated along RDA2 in association with non-regular air filter maintenance and usual multi-passenger occupancy, indicating potential niche preferences unrelated to odor or parking, and instead reflective of cabin maintenance or occupancy habits.

The RDA ordination thus complements and reinforces earlier univariate and GLM findings, showing that moisture perception, parking behavior, and to a lesser extent ventilation and user behavior, structure fungal community composition in vehicles. The distinct distribution patterns across the RDA space highlight species-specific ecological responses to combined environmental and behavioral pressures in enclosed microhabitats such as car interiors.

### 3.3. Relative Abundance Analysis

Analysis of relative species abundance revealed significant links between fungal dominance and vehicle-associated environmental conditions. Kruskal–Wallis tests identified five statistically significant associations (*p* < 0.05) between fungal species and survey variables (Table 1).

*Penicillium* was significantly influenced by parking environment (*p* = 0.0062). Vehicles parked in closed spaces consistently exhibited lower relative abundance of *Penicillium* compared to those parked outside. *Cladosporium* showed a strong inverse association with mold odor perception (*p* = 0.0008). In vehicles where no musty smell was reported, *Cladosporium* presented significantly higher relative abundance, suggesting that *Aspergillus*, rather than *Cladosporium*, is a primary contributor to mold-related odor increases, indicating possible niche differentiation between the two genera. *Rhizopus* also demonstrated a significant association with odor perception (*p* = 0.0003), suggesting a potential role for *Rhizopus* in olfactory cues or its co-occurrence with dominant odor-producing taxa.

*Alternaria* exhibited two distinct and complementary associations: first, relative abundance was significantly higher in vehicles with no regular air filter maintenance (*p* = 0.0075). This highlights the potential of filtration practices to regulate the airborne spore load of *Alternaria* and mitigate colonization risk. Second, *Alternaria* abundance was also elevated in vehicles not parked in closed garages (*p* = 0.026), reinforcing the role of exposure to open air environments as a contributing factor to its prevalence. Collectively, these patterns highlight the ecological specificity of fungal taxa in response to moisture perception, ventilation, and external exposure. The consistent impact of parking environment, odor detection, and air filter maintenance on the proportional representation of dominant species aligns with trends observed in absolute abundance modeling, supporting a robust relationship between environmental behaviors and the composition of fungal communities in vehicle interiors.

## 4. Discussion

To our knowledge, this study provides the first assessment of airborne fungal diversity in private vehicles in Mexico, combining culture-based quantification with user behavior surveys. Airborne fungi are ubiquitous and relevant due to their roles in both ecological distribution and health risk. Our findings align with previous indoor air studies identifying *Cladosporium*, *Aspergillus*, and *Penicillium* as dominant airborne fungi in homes and buildings [5,9,13,14,15,16]. However, studies of vehicle interiors are scarce, and most focus on filter content rather than ambient air [17]. Our findings show a majority of fungi belonging to Ascomycota, with no identified isolate belonging to Basidiomycota, although a recent report on the bioaerosols inside commuter transport showed, by means of sequencing, that Basidiomycota were the predominant fungal group in cars [18]. The methodology employed is fundamental in order to explain this, since most filamentous basidiomycete fungi do not sporulate when cultivated in agar, but until they produce macroscopic fruiting bodies, making it practically impossible to identify by macro- and microscopic features alone, unlike ascomycetous fungi, which produce conidia and conidiophores that can be identified confidently to the genus level [19]. Since the majority of the fungal pathogens relevant to human health are contained in the Ascomycota group, focusing on culturable fungi remains a relevant source of information [4].

The identification of *Bipolaris* as a prevalent taxon is notable, as it is often underreported in closed environments, suggesting vehicle interiors may serve as underrecognized niches for such genera. Regarding *Cladosporium*, a previous report from our group showed that this genus was the most frequently isolated during a year-long sampling campaign, confirming its dominance in Monterrey’s outdoor air [5]. Notably, in contrast to that report and similar studies, the present study revealed lower fungal diversity and an absence of yeasts. This is of interest because although vehicle use patterns could affect fungal concentration and diversity, vehicle cabin air is largely reflective of outdoor air, and given that yeasts are part of human skin and respiratory microbiota, their presence inside vehicles would be expected. A possible explanation for this could be the fact that this study was conducted during the late spring season which in Monterrey represents ambient temperatures of 30 °C and up; parked cars under the sun can quickly exceed 40 °C and reduce humidity, creating an inhospitable environment for yeast survival.

Lower levels of sterile mycelium were observed in undisturbed vehicle air compared to recently entered vehicles. This pattern suggests that passenger activity likely reintroduces and/or resuspends fungal spores settled on surfaces, consistent with findings from studies that highlight human movement as a key factor in indoor particle dynamics [13,20]. This observation supports standardized protocols for air sampling in closed environments, emphasizing the need to consider occupant movement. This is true for many other environments where greater human activity takes place in such environment or where such activity has been more recent, reinforcing the idea that greater amounts of fungal isolations result from spore disturbance rather than an actual greater fungal presence.

Vehicles parked outdoors had significantly higher total fungal loads and *Bipolaris* CFUs than those kept in closed garages. This supports the idea that environmental exposure influences indoor vehicle air quality through infiltration and passive spore deposition [16]. Although *Bipolaris* is a genus commonly found in environmental samples, its presence in our report is unusually high. This may have resulted from very specific local variables or due to a statistical anomaly resulted from the sample size, however, the higher fungal load for outdoor-parked vehicles represents a logical consequence of vehicle use and the study’s seasonal context in which heat and dust charged wind is common. In contrast, covered parking may reduce dust and spore entry reducing both fungal load and organic matter that could be used as nutrient [21].

Vehicles with infrequent air filter replacement exhibited significantly elevated *Alternaria* levels, reinforcing the role of HVAC systems as fungal reservoirs [22]. *Alternaria* spores are abundant in outdoor air, especially in warm, dry seasons, often comprising up to 10% of total airborne fungal spores [23]. These spores readily infiltrate vehicle cabins via open windows or intake vents. Filters capture moisture, organic debris (like pollen and plant fragments), and particulate matter. *Alternaria* thrives on decaying plant matter and organic substrates, making filters an ideal growth environment. The filter’s warm, damp conditions make it suitable for spore germination and turn them into sources of biological contamination [24]. In vehicles, it has been found in up to 85% of filters, along with *Cladosporium*, *Asperillus*, *Penicillium* and *Fusarium*. While *Alternaria* was not the most abundant genus inside vehicles cabins, its increased numbers in vehicles without filter maintenance represents a particular health risk because *Alternaria* is one of the most potent fungal allergens, particularly in children and adolescents. Sensitization to *Alternaria* is strongly correlated with the development of severe, persistent asthma and increased risk of life-threatening asthma attacks and it is considered a “marker allergen” for atopic individuals with higher morbidity. This association is stronger than what is typically seen with *Aspergillus*, *Cladosporium*, or *Penicillium* [25]. Overall, vehicle’s cleaning and maintenance (including air filters), reduces the fungal load inside the vehicle’s cabin [1,5,6,7,15,26].

The perception of musty or moldy odors was positively correlated with *Aspergillus* abundance, with a 2.5-fold increase in CFU levels in odor-reported vehicles. The association of musty/moldy odors (often unpleasant) has been reported abundantly for environments including vehicles and buildings [27,28,29]. While the accuracy of the odor perception vs. fungal presence has not been studied specifically, our report adds to the evidence showing that odor perception is a reliable indicator for fungal presence, especially *Aspergillus* which represents the most important respiratory fungal hazard [30].

The detection of genera such as *Aspergillus*, *Alternaria*, and *Bipolaris* in the air inside vehicle cabins is of concern due to their well-established roles as aeroallergens and opportunistic pathogens. Notably, the significant correlation between musty/moldy odor perception and *Aspergillus* abundance in our samples underscores the potential health risks posed by this genus in confined spaces such as vehicles. *Aspergillus* species are among the most studied airborne fungi, with *A. fumigatus* being a major cause of invasive aspergillosis in immunocompromised individuals, and allergic bronchopulmonary aspergillosis (ABPA) in patients with asthma or cystic fibrosis [31,32]. Moreover, chronic exposure to *Aspergillus* spores has been linked to hypersensitivity pneumonitis and fungal sinusitis, even in otherwise healthy individuals [31]. The presence of this genus in vehicle cabins, as revealed in our study, raises concerns about long-term low-level exposure, particularly in regions with high ambient temperatures and low ventilation exchange rates.

Importantly, *Aspergillus* is listed as a critical priority pathogen in the World Health Organization (WHO) Fungal Priority Pathogens List published in 2022—the highest threat category identified [4]. This designation reflects its global burden, resistance to antifungals, diagnostic challenges, and significant mortality associated with invasive infections. Although not dominant in our samples, *Fusarium* species were sporadically isolated and deserve mention. *Fusarium* is classified by the WHO as a high priority fungal pathogen, due to its ability to cause a spectrum of diseases including keratitis, onychomycosis, and severe invasive fusariosis, particularly in neutropenic patients [33]. Its environmental ubiquity and intrinsic resistance to many antifungal drugs make its presence in indoor or semi-enclosed environments such as vehicle cabins especially concerning.

Collectively, the findings suggest that vehicles can act as underrecognized microhabitats for medically important fungi. Regular vehicle maintenance, particularly air filter replacement and the investigation of mold odors, can reduce fungal load and mitigate potential health risks. This is especially relevant for high-risk populations such as asthmatics, immunocompromised individuals, and professional drivers with prolonged in-cabin exposure.

Although our report included a conservative amount of samples, our multivariate RDA analysis confirmed that specific taxa respond differently to environmental variables. For example, *Aspergillus*, *Alternaria*, and *Penicillium* clustered with odor perception and air filter status, while *Bipolaris* and *Rhizopus* aligned with outdoor parking and moisture exposure. This niche partitioning reflects ecological traits of these fungi, such as sporulation strategies, spore hydrophobicity, and susceptibility to environmental stressors [34]. The distinction between fungi associated with moisture cues and those linked to air exchange or occupancy provides a nuanced understanding of how vehicle environments structure fungal communities. This could provide usefulness in studies of bigger sampling size and those where sampling is carried over large periods of time, offering important data to improve our comprehension regarding fungal presence and ecological distribution.

This study uniquely combines real-time air sampling, detailed behavioral surveys, and multivariate statistics to investigate fungal ecology in vehicles—an approach not commonly reported in the literature. Whereas most previous work has focused on home or occupational indoor environments, our data reveal vehicles as dynamic microenvironments affected by user habits and maintenance, with implications for public health and automotive design.

A suitable improvement of this work should account for seasonal variation, which influence spore abundance [13], incorporate molecular tools to avoid unidentified fungi and include unculturable species and unidentifiable species by morphological means only, and longitudinal sampling.

## Figures and Tables

**Table 1:** Relative abundance associations (Kruskal–Wallis) *.

Species	Variable	*p*-Value
*Penicillium*	Parking environment	0.00617
*Cladosporium*	Musty/moldy odor	0.00076
*Rhizopus*	Musty/moldy odor	0.00034
*Alternaria*	Air filter maintenance	0.00747
*Alternaria*	Parking environment	0.02616

* Only significant results are shown.

## Data Availability

Supporting data can be accessed via https://bit.ly/airfungicars (accessed on 26 May 2025).

## Supplementary Materials

The following supporting information can be downloaded at: https://bit.ly/airfungicars2 (accessed on 26 May 2025).
